# Supplementary figures and images for: The Membrane Activity of the Amphibian Temporin B Peptide Analog TB_KKG6K Sheds Light on the Mechanism That Kills Candida albicans
Source: mSphere. 2022 Aug 16;7(5):e00290-22. doi: 10.1128/msphere.00290-22 (PMC9599520; doi:10.1128/msphere.00290-22)

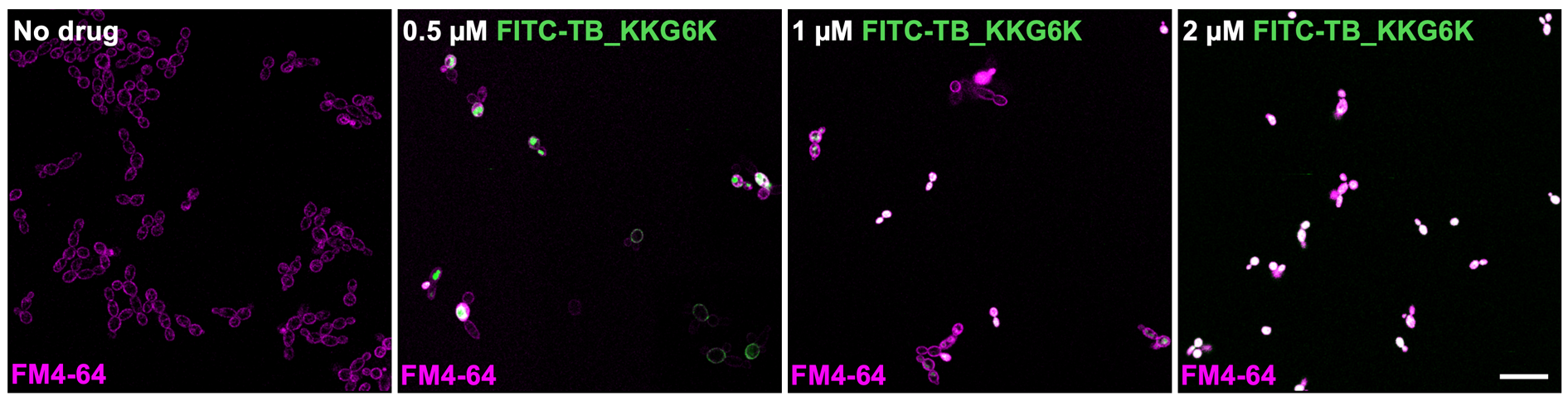

Supplement: FIG S1 [file msphere.00290-22-s0001.tif]

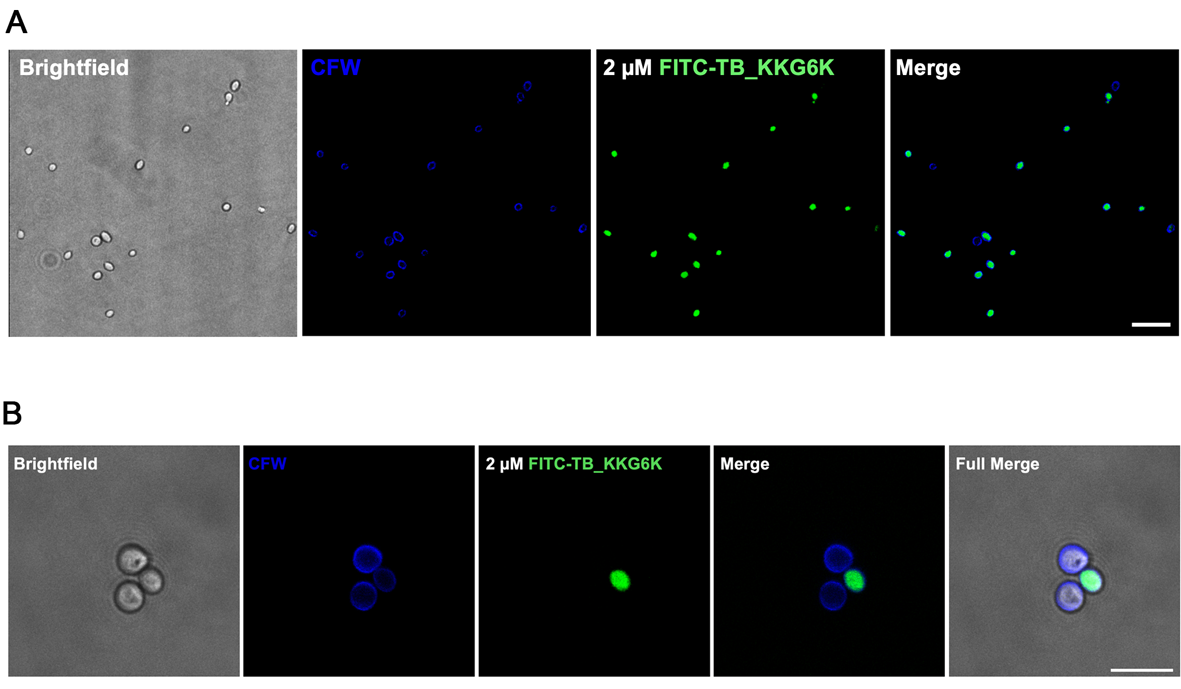

Supplement: FIG S2 [file msphere.00290-22-s0002.tif]
